# Associations of Plasma FGF2 Levels and Polymorphisms in the *FGF2* Gene with Obesity Phenotypes in Han Chinese Population

**DOI:** 10.1038/srep19868

**Published:** 2016-02-16

**Authors:** Ruo-Han Hao, Yan Guo, Shan-Shan Dong, Gai-Zhi Weng, Han Yan, Dong-Li Zhu, Xiao-Feng Chen, Jia-Bin Chen, Tie-Lin Yang

**Affiliations:** 1 Key Laboratory of Biomedical Information Engineering of Ministry of Education, School of Life Science and Technology, Xi’an Jiaotong University, Xi’an 710049, P. R. China; 2 Laboratory of Xi’an Jiaotong University Hospital, Xi’an 710049, P. R. China

## Abstract

Obesity is highly heritable, but the specific genes influencing obesity related traits are largely unknown. Fibroblast growth factor 2 (*FGF2*) could influence adipocyte differentiation. However, the association of *FGF2* polymorphisms and obesity remains unclear. This study aimed to investigate the associations of both the plasma FGF2 levels and SNPs in *FGF2* gene with obesity phenotypes in Han Chinese populations. Plasma FGF2 levels were measured and subjected to association analyses in 62 subjects. Eleven SNPs in *FGF2* were genotyped and tested for associations in a discovery sample of 1,300 subjects. SNPs significantly associated with obesity were subjected to replication in another independent sample of 1,035 subjects. We found that plasma FGF2 levels were positively correlated with fat mass (*P* = 0.010). Association analyses in the discovery sample identified three SNPs (rs1449683, rs167428, rs308442) significantly associated with fat mass after multiple testing adjustments (*P* < 0.0045). Subsequent replication study successfully validated one SNP (rs167428) associated with fat mass (*P*_combine_ = 3.46 × 10^−5^). eQTL analyses revealed that SNPs associated with obesity also affected *FGF2* expression. Our findings suggested that high plasma FGF2 level correlated with increased risk of obesity, and *FGF2* gene polymorphisms could affect individual variances of obesity in Han Chinese population.

Obesity is a major worldwide health problem associated with increased risk of type 2 diabetes, heart diseases, and several forms of cancer^1^. The prevalence of obesity continues to increase at an alarming rate. In China, 32.1% of Chinese are overweight, and 9.9% are obese according to the National Physique Monitoring Bulletin in 2010 (http://www.gov.cn/test/2012-04/19/content_2117320.htm). In the Chinese population, individuals with a BMI ≥ 23 kg/m^2^ are considered as overweight, and those with a BMI ≥ 27.5 kg/m^2^ are obese^2^.

Obesity is a typical complex disease of strong genetic determination, with heritability estimated ranging from 40 to 70%^3^,^4^. In the past years, genome-wide association studies (GWASs) have successfully identified a number of genetic determinants for obesity, such as *FTO*^5^, *INSIG2*^6^, and *MC4R*^6^, etc. However, the identified loci together only account for a small proportion of the genetic predisposition (<10%) to obesity, suggesting that many more associated variants remain to be discovered.

*FGF2* gene is located in chromosome 4q26, and encodes a member of the fibroblast growth factors (FGFs). FGFs are involved in numerous biological processes, including adipogenesis^7^. FGF2, one of the most often investigated cytokines in FGFs, can be synthesized and secreted by human adipocytes^8^. Previous functional studies revealed a close relationship between FGF2 and adipocytes. For example, Kawaguchi *et al.*^9^ found that FGF2 can induce the *de novo* adipogenesis with reconstituted basement membrane supplied, suggesting that FGF2 can influence the distribution of adipose tissue in the body, which in turn affect the risk for metabolic complications. Kakudo *et al.*^10^ discovered that FGF2 significantly enhanced the adipogenic differentiation of adipose-derived stem cells into adipocytes. Given that the major definition of obesity is the excessive proliferation of adipocytes, it reminds us that FGF2 might be related with obesity and *FGF2* gene could be a new candidate gene for obesity. Till now, only one study indicated a negative correlation between serum FGF2 levels and BMI in 30 Japanese overweight men^11^. Recently, Shungin *et al.* has reported a SNP (rs303084) near *FGF2* as a new loci associated with waist-hip ratio adjusting for BMI (WHRadjBMI)^12^, as the only genetic association evidence to reveal the relationship of *FGF2* with obesity. Considering the limited findings, the level of FGF2 and genetic basis in obesity still remain poorly understood. Therefore, the aims of this study were as follows: 1) to evaluate the associations of plasma FGF2 levels with obese status and 2) to investigate the relationship of SNPs in *FGF2* with obesity related traits in Han Chinese populations.

## Results

### Correlation analyses of plasma FGF2 levels with obese status

**Figure 1:**
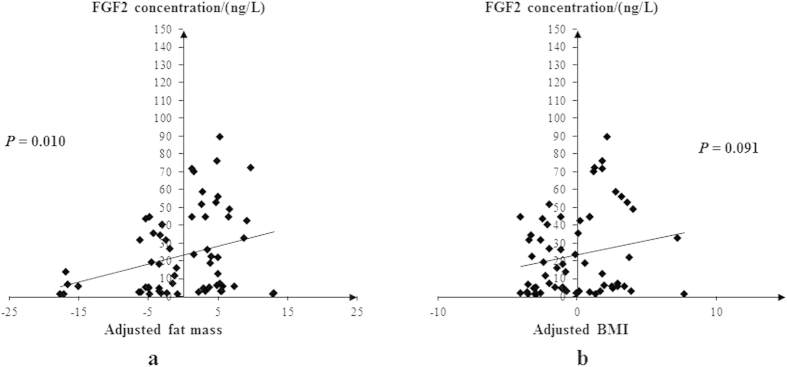
Correlation plots of plasma levels of FGF2 after adjusting for sex and age with (**a**) fat mass and (**b**) BMI.

We performed correlation analyses of plasma FGF2 levels with obesity phenotypes, including BMI and body fat mass, in 62 unrelated Han Chinese subjects. The basic characteristics of this sample are summarized in Table 1. For the plasma sample, the profile of FGF2 level was 23.27 ± 23.39 ng/L, and the correlations between FGF2 level and obesity phenotypes are particularly shown in scatter plots in Fig. 1. Significant positive correlation was observed between FGF2 levels and fat mass (*β* = 0.092 (±0.026), *P* = 0.015), which remained significant even after adjusting for age and sex (*β* = 0.096 (±0.025), *P* = 0.010). However, we did not observe significant association of FGF2 levels with adjusted BMI (*β* = 0.025 (±0.015), *P* = 0.091), even though the association was in the same direction as that with fat mass.

### Association of SNPs in *FGF2* with obesity related traits in the discovery sample

We selected 11 SNPs in *FGF2* to test for associations with obesity-related traits (BMI, fat mass and WHRadjBMI) in the discovery sample of 1,300 Han Chinese subjects. The basic characteristics of this sample are shown in Table 1. SNPs and the major association results are summarized in Table 2. For BMI, only 1 SNP showed nominal significant association (rs167428: *P* = 0.019). For fat mass, 8 SNPs presented nominal significant associations (*P* < 0.05). For WHRadjBMI, SNP rs308387 reached nominal significance (*P* = 0.041). After Bonferroni correction, 3 SNPs remained significant for association with fat mass, including rs1449683 (*P* = 1.11 × 10^−4^), rs167428 (*P* = 1.67 × 10^−4^), and rs308442 (*P* = 5.20 × 10^−4^). Each minor allele of these 3 SNPs was associated with reduced fat mass values with the effect size (*β*) of −0.877 (rs1449683-T), −0.986 (rs167428-C), and −0.724 (rs308442-A), respectively. As for their genetic positions, rs1449683 is located at the exon 1 of *FGF2*, while the other two (rs167428 and rs308442) are close to each other and located at the intron 1. We analyzed the linkage disequilibrium (LD) between rs167428 and rs308442, and found that these two SNPs were in modest LD with each other (pair wise *r*^*2*^ = 0.31, *D*′ = 1.0). We then performed conditional analysis on rs167428 using rs308442 as a covariate to see the independent association effect. The *P* value changed from 0.019 to 0.076 for BMI, and from 1.67 × 10^−4^ to 0.025 for fat mass, respectively. Such results presented significant drop of association signals compared with the regular association analysis, suggesting that the association signals between these two SNPs were highly correlated.

**Figure 2:**
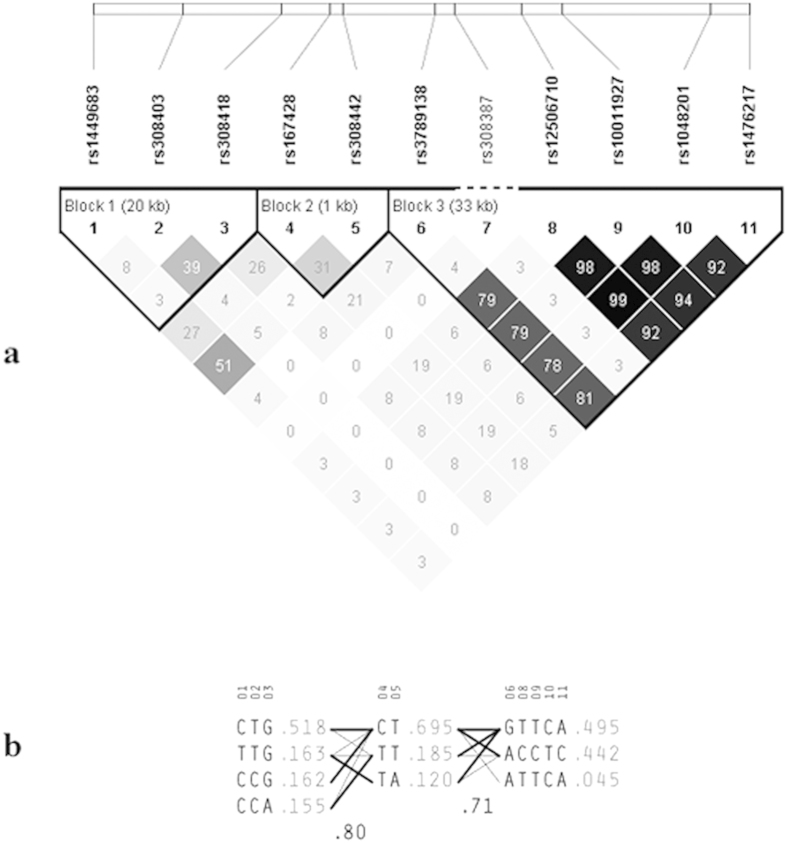
Linkage disequilibrium (LD) blocks and haplotype frequencies for *FGF2* in the discovery sample. (**a**) LD blocks are marked with triangles. Squares color scheme represents the value of *r*^*2*^, and the black color indicates strong LD. (**b**) Haplotypes in the three blocks across *FGF2*. The haplotype frequencies are shown to the right of each haplotype. The SNP numbers correspond to those in Table 2.

We further characterized the LD blocks and haplotypes for SNPs in *FGF2*. As shown in Fig. 2, three blocks in high LD and 10 haplotypes were identified. Nine of these haplotypes were with frequencies more than 0.05 (Fig. 2b). Haplotype association results are listed in Table 3. After multiple testing adjustment, three haplotypes were significantly associated with fat mass, including haplotype “TTG” in block 1 (*P* = 8.71 × 10^−5^, *β* = −0.904), “TA” (*P* = 1.39 × 10^−4^, *β* = −0.999) and “CT” (*P* = 5.20 × 10^−4^, *β* = 0.724) in block 2. Given the 3 significant SNPs included in block 1 (rs1449683) and block 2 (rs167428 and rs308442) separately, haplotype-based association results corroborated the single SNP results.

### Follow-up replication analysis

To further validate the SNP association results, we selected the top 4 significant SNPs (including the three significant SNPs (rs1449683, rs167428, and rs308442) after Bonferroni correction and one SNP rs1048201 located at 3′UTR with nearly significant signal) recognized from the discovery study for replication analyses in another independent Han Chinese sample. However, since the SNP rs1449683 failed genotyping, we had only three SNPs for subsequent analysis. The association results are summarized in Table 4. SNP rs167428 was successfully replicated by showing significant association signal with fat mass (*P* = 0.044) and borderline significant association signal with BMI (*P* = 0.053). The effect was in the same direction as that in the discovery sample. After meta-analysis, the combined *P* value of rs167428 related to fat mass achieved a much more significant level of 3.46 × 10^−5^. We further performed heterogeneity test between our two samples, and no heterogeneity for rs167428 was observed, neither for BMI (*P* = 0.902) nor for Fat Mass (*P* = 0.262).

We also checked the association results for the above SNPs in the published data on GWASs of obesity from the GIANT^12^ consortium and related traits like type 2 diabetes (T2D) from the DIAGRAM^13^ consortium. In the GIANT Consortium dataset consisted of 210,088 European individuals, SNP rs308442 was significantly associated with WHRadjBMI (*P* = 0.024) and hip circumference adjusting for BMI (HIPadjBMI) (*P* = 0.025) in females. SNP rs1048201 was significantly associated with WHRadjBMI (*P* = 0.037) and HIPadjBMI (*P* = 3.8 × 10^−4^) in the total sample, and this SNP was also associated with T2D risk (*P* = 0.01) in the DIAGRAMv3 dataset comprised of 12,171 T2D cases and 56,862 controls of European descent.

### eQTL analysis results

eQTL analysis was performed to investigate the relevance between the identified SNPs and *FGF2* gene expression levels in 210 HapMap individuals. As shown in Table 3, for all the 210 subjects, we identified 5 SNPs (rs1449683, rs167428, rs308442, rs3789138, and rs1048201) significantly associated with *FGF2* mRNA expression (*P*_eQTL_ ≤ 0.01). For rs167428, the effect size (*β*) is −0.059 for minor allele C, with the same direction as genetic association findings. Such observation indicated that this polymorphism could result in the decrease of both FGF2 expression level and the risk of being obese. Moreover, since all the replication tested SNPs (rs167428, rs308442 and rs1048201) passed the eQTL analysis, we further performed association analyses for these three SNPs with plasma FGF2 levels in our 62 Han Chinese subjects. However, no significant signal was observed (rs167428: *P* = 0.133; rs308442: *P* = 0.761; and rs1048201: *P* = 0.462).

## Discussion

Previous studies established FGF2 protein as a positive regulator of human preadipocytes proliferation and differentiation^14^. For *in vivo* evidence, injection of FGF2 and basement membrane in mice resulted in *de novo* adipogenesis^9^. Given the biological function of FGF2 protein involved in adipocytes, it is reasonable to hypothesize that FGF2 could influence adipose-related trait.

To test the hypothesis, firstly, we performed association tests to determine whether plasma FGF2 was correlated with obesity. We found that plasma FGF2 levels were positively correlated with fat mass, and marginally correlated with BMI in the same direction, suggesting that FGF2 might play a positive role in obesity. However, a previous study reported that serum FGF2 levels were negatively correlated with BMI, visceral fat and subcutaneous fat in a 30 Japanese population^11^. Such discrepancy might be due to the population specificity and the relatively small sample size of that study. Then, we hypothesized that association might also exist between the SNPs in *FGF2* gene and obesity traits. Given the limited evidence of association study for human *FGF2* gene with obesity, we investigated whether the common variants in *FGF2* were associated with obesity phenotypes, including BMI, fat mass and WHRadjBMI. We identified one SNP (rs167428) significantly associated with fat mass both in our Chinese discovery and replication samples. Previous study by Shungin *et al.* reported a SNP (rs303084) near *FGF2* as a new loci associated with WHRadjBMI^12^. This SNP was over 200kb far away from *FGF2*. We examined LD between our identified SNP and the reported SNP using SNAP (data were derived from the CHBJPT panel of HapMap 3 (release 2) dataset)^15^. The pairwise LD was quite weak (*r*^*2*^ = 0.010, *D*′ = 0.207), suggesting that our identified SNP could be a novel independent loci for obesity. To further assess the functional role of the identified SNPs, we performed eQTL analyses and found that SNPs associated with fat mass also affected mRNA expression level of *FGF2* in human LCLs using public HapMap data. Taking into account of all those biological evidence and our statistical findings, we suggested that *FGF2* gene could have potential impact on human fat mass regulation, and high plasma FGF2 level might lead to the increased risk of obesity.

Using the public available datasets on GWASs of obesity related traits, we sought for further evidence to support our findings. In the GIANT Consortium, although the SNP rs167428 was not validated, another SNP, rs308442, which was in modest LD with rs167428 (*r*^*2*^ = 0.31, *D*′ = 1.0) and belonged to one block, was found to be associated with HIPadjBMI and WHRadjBMI in females. Moreover, a SNP (rs1048201) in 3′UTR of *FGF2* was found to be associated with HIPadjBMI and WHRadjBMI, and also associated with T2D risk in the DIAGRAM Consortium. These two SNPs showed significant signals in our Chinese discovery sample. Since the subjects in the two large consortiums are all European ancestry individuals, the different LD patterns and population structures might cause the discrepancy of associated SNPs. However, all of these significant evidences, combined with our findings, support *FGF2* as an obesity-associated gene in humans.

Our study samples were recruited from Midwestern area of China, where is relatively conservative and contains homogenous population. Using the same population, our previous published GWAS studies^16^,^17^ demonstrated that there was no population stratification in this population. Therefore, our association results are unlikely to be plagued by spurious associations due to population stratification.

Our study used quantitative obesity-phenotypes to test for genetic associations. When taking obesity as categorical outcome based on BMI (people who had the BMI > 27.5 kg/m^2^ were considered obese according to the universal classification criterion of obesity of Asian), there were only 67 (5.2%) and 100 (9.7%) obese individuals in the discovery and replication cohorts, respectively. Since limited sample size with low statistical power might reduce the chance of detecting a true effect, analysis for association with obesity as categorical trait was not preferred in our study.

In our study, we did not find significant association with BMI. This may be due to the incomprehensive estimation of obesity by BMI. BMI is based on the observation that body weight is proportional to the squared height in adults with normal body frames^18^. This property makes BMI ignoring several important factors affecting adiposity. For instance, individuals with a normal BMI may suffer from sarcopenic obesity due to greater loss of muscle mass^19^. Fat mass derived from DXA, on the other side, is a direct measurement of fat, is more homogeneous and may reflect obese status more accurately. Therefore, fat mass can characterize obesity from different aspects and could be used as a complementary indicator for obesity.

In conclusion, our study provided novel evidence that human plasma FGF2 levels were positively correlated with fat mass, and genetic polymorphisms in *FGF2* gene were associated with fat mass in Han Chinese population. Our results suggest that *FGF2* could be a novel candidate for obesity by processing a series of association tests and referring to its biological functions. The evidence of the biological mechanism of FGF2 in body fat mass regulation is needed to confirm our findings in later studies.

## Materials and Methods

### Subjects

The study was approved by the Institutional Review Board of Xi’an Jiaotong University and carried out in accordance with the approved guidelines. All the volunteers signed informed consent documents before entering the study.

There were three sample sets in our study. For the plasma FGF2 levels test, we included 62 unrelated subjects. For the SNP association test, we expanded our sample size to 1,300 subjects as the discovery phase, and an additional 1,035 subjects as the replication phase, which contained the 62 plasma sample subjects. All the subjects were unrelated northern Han Chinese adults that were recruited from the city of Xi’an and its neighboring areas. Subjects with chronic diseases and conditions that might potentially affect body mass, structure, or metabolism were excluded from the study to minimize the influence of known environmental and therapeutic factors on BMI and fat mass variation. These diseases/conditions included chronic disorders involving vital organs (heart, lung, liver, kidney, and brain), serious metabolic diseases (diabetes, hypo- and hyper-parathyroidism, hyperthyroidism, etc.), chronic use of drugs affecting body mass (hormone replacement therapy, corticosteroid therapy, anti-convulsant drugs), and malnutrition conditions (such as chronic diarrhea, chronic ulcerative colitis, etc.), etc.

### Phenotype measurements

Waist and hip circumferences were measured with a calibrated tape measure. BMI values were calculated as body weight (in kilograms) divided by the square of height (in meters). Weight was measured in light indoor clothing without shoes, using a calibrated balance beam scale, and height was measured using a calibrated stadiometer. Body fat mass (in kilograms) was measured using dual energy X-ray absorptiometry (DXA) by Hologic 4500 W machines (Hologic Inc., Bedford, MA, USA) under strict protocols. The coefficient of variation (CV) value of the DXA measurement for fat mass was approximately 1.1%. In this study, we included BMI, body fat mass and WHRadjBMI, to assess obese status^12^,^20^.

### Plasma FGF2 concentrations measurement

Fasting blood samples were obtained in the morning from 8:00 to 10:00 AM. Plasma samples were collected by blood separation with centrifugation at 2,500 rpm for 10 min at 4 °C, following both the manufacturer’s protocol and specialized laboratory assay quality control procedures, and the samples were saved at −80 °C until analysis (no longer than 6 months). FGF2 concentrations were measured through enzyme-linked immunosorbent assay (ELISA) using ELISA commercial kit (RapidBio, West Hills, CA, USA) strictly under the manufacturer’s protocol. According to the instruction, the plate coefficient of variation was less than 15%, the assay range was 1.0 to 100.0 ng/L, and the sensitivity was less than 1.0 ng/L. No soluble structural analogues with other cross-reaction were observed during the whole procedure.

### SNP selection and genotyping

Genomic DNA was extracted from peripheral blood leukocytes using a commercial isolation kit (Gentra systems, Minneapolis, MN, USA) following the protocol of the kit. We explored the dbSNP (http://www.ncbi.nlm.nih.gov/SNP) and HapMap (http://hapmap.ncbi.nlm.nih.gov/cgi-perl/gbrowse/hapmap28_B36/) databases searching SNPs in *FGF2*. We selected 11 SNPs spanned the genic region in *FGF2* according to the following criteria, in order of importance in our selection scheme: (i) minor allele frequency (MAF) > 0.05 in the CHB population; (ii) an average density of 1 SNP per 7 kb; (iii) tagging SNPs are preferential; (iv) potential functional SNPs.

For the discovery sample, SNPs genotyping was carried out using MALDI-TOF mass spectrometry on a MassARRAY system (Sequenom, Inc., San Diego, CA) with iPLEX assay. Genotype calling was performed in real time with MassARRAY RT software version 3.0.0.4 and analyzed using the MassARRAY Typer software version 3.4 (Sequenom). Genotyping quality control procedures leading to SNP exclusion were call rate < 90%, MAF < 0.05 and *P* < 0.001 for deviations from Hardy–Weinberg equilibrium (HWE). The average call rate was 98% and the duplicate concordance rate was 99%. All of the 11 SNPs in the discovery sample were successfully genotyped and used for association analyses. Then, we selected the top 4 significant SNPs in the discovery phase to perform replication study. SNPs genotyping used in the replication sample was the same as that adopted in the discovery sample. Of the 4 SNPs attempted, one SNP (rs1449683) failed in the replication genotyping procedure.

### Statistical analyses

The raw BMI and fat mass values were adjusted with covariates including sex and age. The residuals were used for further association analyses. For the plasma FGF2 analyses, we used linear regression model to assess the relationship of plasma FGF2 concentrations with obese status and genetic variants separately. These procedures were conducted with Minitab 14 software (PA, USA). For the SNP association analyses, linear regression implemented in PLINK^21^ was fitted to test for associations under the additive inheritance model in the discovery sample and the replication sample respectively. Population haplotypes and their frequencies were inferred using Haploview software^22^. Heat plot of linkage disequilibrium using *r*^*2*^ value was also generated by Haploview^22^. Haplotypes with estimated frequencies greater than 5% were included for association analyses by PLINK^21^. A raw *P* value of < 0.05 in our study was considered nominally significant, and the Bonferroni correction was used for multiple testing corrections. Finally, the significance threshold was set as *P* < 0.0045 in the discovery sample for single SNP test (0.05/11 SNPs that were included in the association analyses) and *P* < 0.0056 for haplotype analysis (0.05/9 haplotypes that were included in the association analyses).

Meta-analysis statistics and heterogeneity test were generated using METAL software package (http://genome.sph.umich.edu/wiki/METAL_Documentation). The meta-analyses were performed with the inverse of the corresponding standard errors weighted effect size, and the Cochran’s *Q*-test was implemented for heterogeneity.

### Expression quantitative trait locus (eQTL) analysis

To explore whether the identified SNPs correlated with the transcription variants, we conducted association analysis between SNPs and mRNA expression levels of the *FGF2* gene. Gene expression data were derived from human lymphoblastoid cell lines (LCLs) of 210 unrelated individuals from HapMap populations in the NCBI Gene Expression Omnibus^23^,^24^. The sample comprised 60 CEU, 45 CHB, 45 JPT and 60 YRI. SNP genotype data were derived from the corresponding HapMap Phase III dataset. The associations between expression levels and SNPs were examined using PLINK^21^ through linear regression model.

## Additional Information

**How to cite this article**: Hao, R.-H. *et al.* Associations of Plasma FGF2 Levels and Polymorphisms in the *FGF2* Gene with Obesity Phenotypes in Han Chinese Population. *Sci. Rep.* **6**, 19868; doi: 10.1038/srep19868 (2016).

## Figures and Tables

**Table 1:** Basic characteristics of the study samples.

Trait	Plasma Sample	Discovery Sample	Replication Sample
Number	62	1,300	1,035
Male/Female	18/44	600/700	345/690
Age (years)	63.08 (6.84)	33.42 (11.32)	50.36 (17.75)
Weight (kg)	62.40 (9.98)	59.63 (10.41)	61.72 (10.32)
Height (cm)	159.94 (7.77)	163.94 (8.11)	161.85 (7.99)
BMI (kg/m^2^)	24.31 (2.81)	22.11 (2.98)	23.53 (3.28)
Fat Mass (kg)	19.53 (7.04)	13.99 (5.38)	16.21 (7.10)
Hip circumference (m)	—	0.92 (0.06)	0.96 (0.07)
Waist circumference (m)	—	0.75 (0.09)	0.79 (0.11)
Waist-hip ratio	—	0.80 (0.07)	0.82 (0.08)
Plasma FGF2 (ng/L)	23.27 (23.39)	—	—

Note: Normally distributed data are shown as mean (standard deviation, SD).

**Table 2:** Association results for 11 SNPs in *FGF2* with BMI, fat mass and WHRadjBMI in the Chinese discovery sample.

No.	SNP	Physical Position	Genic Position	A1/A2^a^	MAF	BMI		Fat Mass		WHRadjBMI		*β*_eQTL_^b^	*P*_eQTL_^b^
						*β*	*P*	*β*	*P*	*β*	*P*		
1	**rs1449683**	123748086	Exon1	T/C	0.165	−0.255	0.061	−0.877	**1.11 × 10**^**−4**^	−3.03 × 10^−3^	0.261	−0.059	4.86 × 10^−3^
2	rs308403	123757748	Intron1	C/T	0.320	0.170	0.140	0.251	0.193	1.54 × 10^−3^	0.501	—	—
3	rs308418	123768263	Intron1	A/G	0.157	0.139	0.340	0.124	0.610	5.38 × 10^−4^	0.852	−0.030	0.288
4	**rs167428**	123773439	Intron1	C/T	0.120	−0.367	0.019	−0.986	**1.67 × 10**^−**4**^	−3.10 × 10^−3^	0.321	−0.059	2.05 × 10^−5^
5	**rs308442**	123774913	Intron1	A/T	0.305	−0.202	0.107	−0.724	**5.20 × 10**^−**4**^	−1.21 × 10^−3^	0.625	−0.065	1.40 × 10^−6^
6	rs3789138	123784721	Intron1	G/A	0.500	−0.127	0.212	−0.377	0.027	1.66 × 10^−3^	0.412	0.070	5.09 × 10^−6^
7	rs308387	123786832	Intron1	A/G	0. 043	−0.115	0.651	−0.299	0.482	−1.03 × 10^−2^	0.041	—	—
8	rs12506710	123794127	Intron1	C/T	0.445	0.157	0.122	0.419	0.013	−9.34 × 10^−5^	0.963	—	—
9	rs10011927	123798428	Intron2	C/T	0.449	0.155	0.127	0.427	0.011	5.77 × 10^−5^	0.977	—	—
10	rs1048201	123814308	3′UTR	T/C	0.447	0.173	0.089	0.450	7.92 × 10^−3^	−2.06 × 10^−4^	0.919	0.046	0.010
11	rs1476217	123818511	3′UTR	C/A	0.452	0.157	0.135	0.432	0.013	3.44 × 10^−4^	0.869	−0.016	0.331

Note: MAF, minor allele frequency. *β* is calculated with respect to the minor allele. Significant *P* values after multiple testing adjustment (*P* < 0.0045) are shown in bold.

^a^A1 represents the minor allele.

^b^*β*_eQTL_ and *P*_eQTL_ are shown for 210 HapMap unrelated individuals from 4 populations. *β*_eQTL_ is calculated with respect to the minor allele.

**Table 3:** Haplotype identification and association analyses in the discovery sample.

Block	SNPs^a^	Haplotype^b^	Frequency	BMI		Fat Mass		WHRadjBMI	
				*β*	*P*	*β*	*P*	*β*	*P*
Block 1	1–3	CCA	0.155	0.144	0.328	0.132	0.592	1.03 × 10^−3^	0.723
		CCG	0.162	0.132	0.374	0.273	0.270	1.62 × 10^−3^	0.579
		**TTG**	0.162	−0.268	0.053	−0.904	**8.71 × 10**^−**5**^	−4.36 × 10^−3^	0.109
		CTG	0.517	0.018	0.871	0.348	0.055	1.20 × 10^−3^	0.574
Block 2	4,5	**TA**	0.120	−0.374	0.017	−0.999	**1.39 × 10**^−**4**^	−4.23 × 10^−3^	0.172
		TT	0.185	0.048	0.745	−0.127	0.605	1.73 × 10^−3^	0.548
		**CT**	0.695	0.202	0.107	0.724	**5.20 × 10**^−**4**^	1.41 × 10^−3^	0.566
Block 3	6,8–11	ACCTC	0.440	0.167	0.103	0.440	9.82 × 10^−3^	−1.71 × 10^−4^	0.932
		GTTCA	0.495	−0.121	0.237	−0.376	0.027	2.09 × 10^−3^	0.299

Significant *P* values after multiple testing adjustment (*P* < 0.0056) are shown in bold.

^a^The IDs of SNPs correspond to those in Table 2.

^b^Haplotypes with estimated frequencies more than 0.05 are listed.

**Table 4:** Association results of 3 tested SNPs with obesity-related traits in the replication sample, GIANT and DIAGRAM Consortiums.

			BMI				Fat Mass				WHRadjBMI				GIANT^b^		
SNP	A1/A2^a^	MAF	*β*	*P*	*β*_meta_	*P*_meta_	*β*	*P*	*β*_meta_	*P*_meta_	*β*	*P*	*β*_meta_	*P*_meta_	WHRadjBMI	HIPadjBMI	DIAGRAM^c^
rs167428	C/T	0.110	−0.385	0.053	−0.374	2.48 × 10^−3^	−0.684	0.044	−0.888	3.46 × 10^−5^	−3.34 × 10^−3^	0.402	−3.90 × 10^−3^	0.111	0.44	0.15	0.29
rs308442	A/T	0.196	−0.083	0.595	−0.156	0.111	−0.099	0.710	−0.517	2.38 × 10^−3^	−2.57 × 10^−3^	0.414	−1.90 × 10^−3^	0.339	0.19	6.8 × 10^−2^	0.18
rs1048201	T/C	0.442	−0.261	0.040	0.005	0.951	−0.481	0.044	0.139	0.314	−1.56 × 10^−3^	0.533	−1.00 × 10^−3^	0.529	0.037	3.8 × 10^−4^	0.01

MAF, minor allele frequency. *β* is calculated with respect to the minor allele. Meta-analysis was performed with effect size estimates weighted by the inverse of the corresponding standard errors (*β*meta and *P*meta).

^a^A1 represents the minor allele.

^b^WHRadjBMI and HIPadjBMI association *P* values are from the GIANT Consortium of 210,088 European individuals.

^c^*P* values are from the DIAGRAM Consortium of 12,171 T2D cases and 56,862 controls of European descent.
